# Systemic lupus erythematosus with diffuse splenic calcifications: a rare combination

**DOI:** 10.1590/0100-3984.2017.0109

**Published:** 2018

**Authors:** Guilherme Felix Louza, Miriam Menna Barreto, Gláucia Zanetti, Edson Marchiori

**Affiliations:** 1 Universidade Federal do Rio de Janeiro (UFRJ), Rio de Janeiro, RJ, Brazil.

**Figure 1:**
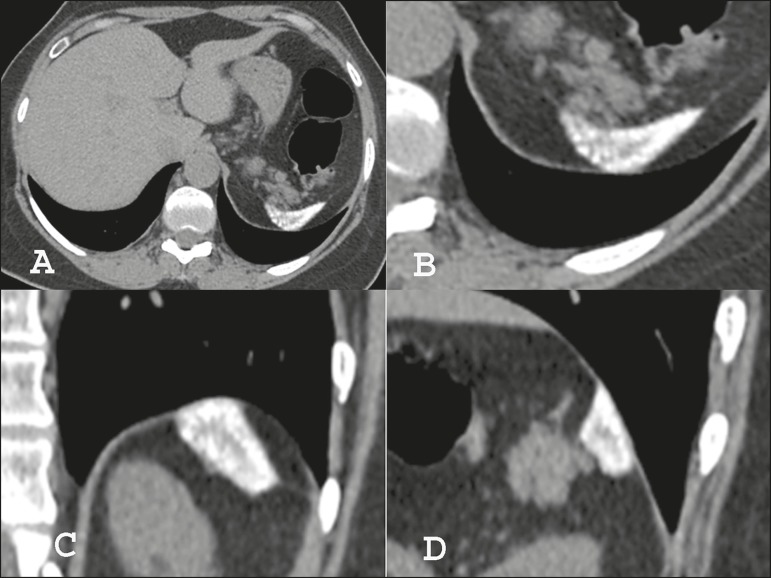
Computed tomography of the chest with mediastinal window settings (**A**) showing a decreased spleen size, accompanied by diffuse, small, predominantly subcapsular and peripheral, nodular calcifications, some of them confluent, with relative sparing of the central regions. Detail of the splenic region: axial computed tomography scan (**B**), with coronal and sagittal reconstructions (**C** and **D**, respectively), showing the characteristics of the splenic calcifications in more detail.

Dear Editor,

We report a case of 46-year-old white female with a history of systemic lupus erythematosus (SLE), diagnosed 19 years prior, who had previously been hospitalized for lupus myocarditis and class IV lupus nephritis. She was currently under treatment with hydroxychloroquine and prednisone. She reported sporadic arthralgia, with relief after analgesic use. She reported no history of infectious diseases. Transthoracic echocardiography revealed normal systolic and diastolic function, with mild aortic, tricuspid, mitral, and pulmonary valve regurgitation. Laboratory tests showed positivity for anti-double stranded DNA antibody and anti-single stranded DNA antibody, with a decreased CH50 (7 U/mL; reference range: 23.0-46.0 U/mL), C3 (47 mg/L; reference range: 90-180 mg/L), and C4 (< 6 mg/L; reference range: 10-40 mg/L). Urinalysis showed normal urinary creatinine (624 mg/24 h; reference range: 0.6-1.6 g/24 h) and elevated urinary protein (232 mg/24 h; reference range: < 150 mg/24 h). During the follow-up of the patient, an abdominal ultrasound was requested, and, during the examination, the spleen could not be visualized, although no other abnormalities. To rule out SLE-related systemic abnormalities, whole-body computed tomography was performed. The examination showed decreased spleen size, accompanied by small, diffuse, predominantly subcapsular and peripheral, nodular calcifications, some of which were confluent, with relative sparing of the central regions (Figure 1).

SLE is a chronic multisystem autoimmune disease, in which a variety of organs and tissues are damaged by pathogenic auto-antibodies and immune complexes^(^^1^^)^. Abdominal involvement of SLE can occurs in virtually any organ within the abdominal cavity (peritoneum, gastrointestinal tract, pancreas, kidney, adrenal gland, hepatobiliary tract, or spleen), although only renal involvement integrates diagnostic criteria^(^^1^^)^. Splenic involvement in SLE is rare. Splenomegaly, splenic infarcts, spontaneous rupture, functional asplenia, hyposplenism and periarterial thickening in an “onion-skin” pattern have all been reported in SLE patients^(^^2^^,^^3^^)^.

Splenic calcifications have been described in a myriad of other diseases, including tuberculosis, histoplasmosis, brucellosis, amyloidosis, sickle cell anemia, anthracosilicosis, systemic sclerosis, and rheumatoid arthritis^(^^3^^,^^4^^)^. Based on the clinical history, physical examination, and laboratory findings, those potential causes of diffuse splenic calcifications were excluded in our case. Tieng et al.^(^^4^^)^ proposed that diffuse splenic calcifications that are predominantly discrete, rounded, and small (although larger than the punctuate calcifications typical of granulomatous infections), as well as appearing to spare the capsule and subcapsular tissue, seem to be specific for SLE. This pattern may represent calcifications in the typical splenic “onion-skin” pattern (i.e., concentric deposition of collagen around the arteries in the spleen) in SLE^(^^2^^-^^4^^)^. Splenic microcalcifications could represent a late consequence of immune-mediated inflammation of arterial vessels^(^^3^^)^.

In conclusion, we have reported the case of a female patient with decreased spleen size and diffuse small nodular calcifications, showing subcapsular and peripheral predominance, with relative sparing of central regions, an atypical distribution in comparison to cases of SLE-related spleen calcifications reported in the literature.
